# Supplementary material for: Medium-Term Effects of Sprinkler Irrigation Combined with a Single Compost Application on Water and Rice Productivity and Food Safety
Source: Plants (Basel). 2023 Jan 19;12(3):456. doi: 10.3390/plants12030456 (PMC9919156; doi:10.3390/plants12030456)
Supplement: Supplementary file 1 [file plants-12-00456-s001.zip › plants-2142832-supplementary.pdf]

Supplementary Material "Medium-term effects of sprinkler irrigation combined with a single compost application on water and rice productivity and food safety"

Table S1. Effects of different management systems on soil physicochemical properties (0-20cm depht).

	**TOC ($g\ kg^{-1}$)**	**FA ($g\ kg^{-1}$)**	**HA ($g\ kg^{-1}$)**	**EC ($dS\ m^{-1}$)**	**pH**	**N ($g\ kg^{-1}$)**	**P ($mg\ kg^{-1}$)**	**AS (%)**
2018								
SNT	11.1aA	0.559aA	0.869aA	1.45aA	6.73dB	1.16aA	36.9aA	56.5bA
SNT-C	16.8bA	1.05dA	1.53bA	1.79bA	6.94eB	1.77bA	48.5dA	50.9abA
ST	10.2aA	0.891bA	0.911aA	1.87bA	6.27cA	1.10aA	51.2eB	43.6aA
ST-C	16.6bA	1.25eA	1.41bA	2.08cA	6.89eB	1.88bA	46.2cA	51.4abA
FT	10.8aB	0.831bA	0.858aA	1.93bA	5.52aA	1.23aA	40.2bA	53.7abA
FT-C	20.9cA	0.971cA	1.48bA	1.52aA	5.92bA	2.07cA	41.4bB	68.4cA
2019								
SNT	12.3cA	0.775aB	0.958bA	5.97dB	6.46dA	1.35aB	41.4abA	72.3bcdB
SNT-C	21.6eB	1.04bA	1.96dA	3.73cB	6.61eA	2.20dB	57.8cB	81.6dB
ST	11.6bB	1.00bA	0.736aA	2.12aA	6.29cA	1.32aA	40.3abA	63.7abB
ST-C	18.6dB	1.18cA	1.30cA	3.93cB	6.62eA	1.84bA	44.1bA	67.8abcB
FT	10.1aA	0.939bA	0.833abA	2.74bB	5.64aB	1.35aA	35.2aA	58.1aA
FT-C	21.4eA	0.996bA	1.44cA	2.65bB	6.11bB	2.03cA	38.2abA	78.1cdA
Y	***	*	NS	***	***	***	NS	***
T	***	***	***	***	***	***	***	**
Y x T	**	NS	*	***	***	**	***	*

TOC: Total Organic Carbon; FA: Fulvic Acid; HA: Humic Acid; EC: Electrical Conductivity; N: Total Nitrogen; P: Available Phosphorus; AS: Aggregate stability. ANOVA factors are Y: Year; T: Treatment; Y x T: Interaction Year * Treatment. F-values indicate the significance levels * $p<0.05$; ** $p<0.01$; *** $p<0.001$, respectively, and NS: not significant. Different letters indicate differences ($p<0.05$) between treatments in the same year (lower case letters) and between years within the same treatment (upper case letters)

The soil physicochemical properties were analysed as described Cabrera et al. (2010).

Cabrera, D.; López-Piñeiro, A.; Albarrán, A.; Peña, D. Direct and residual effects on diuron behaviour and persistence following two-phase olive mill waste addition to soil: Field and laboratory experiments. Geoderma 2010, 157, 133–141. https://doi:10.1016/j.geoderma.2010.04.004.

Table S2. Properties of compost used in the experiment.

Properties	Value
pH	7.71
EC (dS m^{-1})	2.32
TOC (g kg^{-1})	382
N_T (%)	2.17
C/N	17.6
As (mg kg^{-1})	4.00
Cd (mg kg^{-1})	0.148

EC: Electrical Conductivity; TOC: Total Organic Carbon; N: Total Nitrogen

Table S3. Mean maximum (Max) and minimum (Min) temperatures, rainfall and rice evapotranspiration (ETc), registered at the field location during 2018 and 2019

2018				
**Month**	**Max Temp (°C)**	**Min Temp (°C)**	**Rainfall (mm)**	***ETc* (mm)**
January	13.3	3.20	43.4	-
February	15.1	1.22	24.7	-
March	15.8	6.11	173	-
April	19.9	7.91	78.2	-
May	23.4	9.43	36.8	125
June	28.7	13.9	12.6	169
July	31.0	14.8	0	223
August	36.4	17.6	0	224
September	32.8	16.4	18.3	122
October	27.3	10.1	27.7	-
November	16.7	6.37	78.8	-
December	13.0	2.22	17.7	-
2019				
**Month**	**Max Temp (°C)**	**Min Temp (°C)**	**Rainfall (mm)**	***ETc* (mm)**
January	14.1	0.08	28.5	-
February	17.6	1.99	17.9	-
March	20.8	3.51	22.3	-
April	20.7	7.22	38.8	-
May	27.9	10.4	6.0	143
June	28.8	12.1	1.41	175
July	32.7	15.8	1.41	241
August	33.7	15.9	0	202
September	30.7	14.1	8.84	123
October	28.5	11.5	7.84	-
November	17.1	8.96	82.6	-
December	16.0	6.21	79.4	-

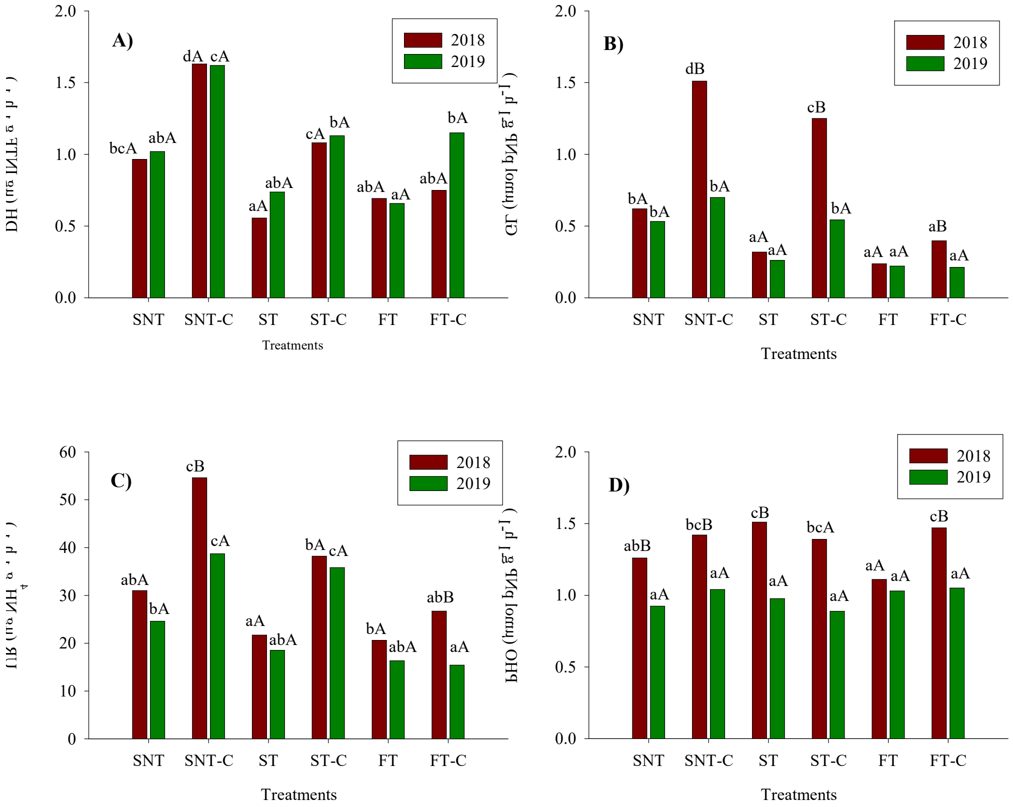

Figure S1. Effects of different management systems on dehydrogenase activity (A), B-glucosidase (B), Urease (C) and Phosphatase (D) (0-10cm depht). Different letters indicate differences ($p<0.05$) between treatments in the same year (lower case letters) and between years within the same treatment (upper case letters).

Dehydrogenase (DH), β-glucosidase (GL), Urease (UR) and Phosphatase (PHO) were determined in the soils samples as described by López-Piñeiro et al. (2011).

López-Piñeiro, A.; Albarrán, A.; Rato-Nunes, J.M.; Peña, D.; Cabrera, D. Long-term impacts of de-oiled two-phase olive mill waste on soil chemical properties, enzyme activities and productivity in an olive grove. Soil Tillage Res. 2011, 114, 175-18. https://doi:10.1016/j.still.2011.05.002.
